# The Global Burden of Air Pollution on Mortality: Anenberg et al. Respond

**DOI:** 10.1289/ehp.1003276R

**Published:** 2011-04

**Authors:** Susan C. Anenberg, J. Jason West, Larry W. Horowitz, Daniel Q. Tong

**Affiliations:** The University of North Carolina at Chapel Hill, Chapel Hill, North Carolina, E-mail: jasonwest@unc.edu; NOAA Geophysical Fluid Dynamics Laboratory, Princeton, New Jersey; Science and Technology Corporation at NOAA Air Resources Laboratory, Silver Spring, Maryland

Prueitt and Goodman raise concerns about our use of chronic ozone mortality relative risk (RR) estimates from Jerrett et al. (2009) to estimate the global burden of outdoor ozone and fine particulate matter (< 2.5 μm in aerodynamic diameter; PM_2.5_) on human mortality (Anenberg et al. 2010). We believe that our use of RR estimates from Jerrett et al. (2009) is justified and does not strongly affect our conclusions. Our goal of demonstrating the use of chemical transport models in estimating the global burden of outdoor air pollution on mortality is not affected by the choice of risk estimates. Further, using chronic RR estimates for ozone has only a minor effect on our mortality estimates, because the mortalities attributed to PM_2.5_ are much greater than those for ozone.

We chose to use RR estimates from Jerrett et al. (2009) in our study (Anenberg et al. 2010) because they are consistent with the widely accepted RR estimates used for long-term PM_2.5_ mortality (Krewski et al. 2009), as both are based on the American Cancer Society study cohort and capture delayed mortality effects (National Research Council 2008).

In response to particular criticisms, we note that while Jerrett et al. (2009) found the first significant positive association between chronic ozone exposure and mortality in a major cohort study, some previous smaller cohort studies have also found positive associations (National Research Council 2008). Biological plausibility for chronic ozone effects on respiratory mortality is evidenced by toxicology and human exposure studies that found that ozone affects airway inflammation, pulmonary function, and asthma induction and exacerbation (National Research Council 2008). Using earlier PM_2.5_ data would be unlikely to affect confounding in the model, because using PM_2.5_ data from 1979–1983 and 1999–2000 yields similar PM_2.5_ mortality associations (e.g., Krewski et al. 2009). Jerrett et al. (2009) also found that socioeconomic data are not strong confounders and that using more recent data is unlikely to change that conclusion (see Appendix of Jerrett et al. 2009). Finally, national risk estimates are more applicable globally than city-specific estimates because they include larger and more diverse populations.

However, because the evidence for chronic ozone mortality is more limited than the large body of evidence demonstrating mortality associations with short-term ozone exposure, we present here estimates of the global burden of ozone on mortality using RR estimates from Bell et al. (2004), a large multicity study of short-term ozone mortality. We estimated mortalities daily using the difference between preindustrial and present-day 8-hr maximum ozone, and sum mortalities over the 1-year simulation. We used the reported relationship for cardiopulmonary mortality and daily average ozone [0.64% (95% posterior interval, 0.31–0.98%) for a 10-ppb increase], and corrected to 8-hr ozone using the reported ratio between daily 8-hr and 24-hr average ozone associations with nonaccidental mortality.

Using these methods, we estimated 362,000 (95% confidence interval, 173,000–551,000) annual global premature cardiopulmonary deaths attributable to ozone, approximately 50% of the 700,000 premature deaths we calculated in our original study (Anenberg et al. 2010). Since estimated deaths due to PM_2.5_ (3.7 million) are an order of magnitude larger, using a short-term rather than long-term RR estimate for ozone has only a minor effect on the overall global burden of disease due to outdoor air pollution. As RRs for chronic ozone mortality are not as strongly supported as those for PM_2.5_, we expect that estimates of mortality burden will improve as research on chronic ozone exposure and mortality continues globally.
